# Recent Advances in Management of Neuropathic, Nociceptive, and Chronic Pain: A Narrative Review with Focus on Nanomedicine, Gene Therapy, Stem Cell Therapy, and Newer Therapeutic Options

**DOI:** 10.1007/s11916-024-01227-5

**Published:** 2024-02-22

**Authors:** Saurabh Kataria, Utsav Patel, Kevin Yabut, Jayshil Patel, Rajkumar Patel, Savan Patel, Jeremiah Hilkiah Wijaya, Pankti Maniyar, Yukti Karki, Moinulhaq P. Makrani, Omar Viswanath, Alan D. Kaye

**Affiliations:** 1 Department of Neurology, Louisiana State University Health Sciences Center at Shreveport, Shreveport, LA 71103 USA; 2 LSU Health Science Center at Shreveport, 1501 Kings Highway, Shreveport, LA 71104 USA; 3 Mayo Clinic, Jacksonville, FL 32224 USA; 4 Louisiana State University Health Science Center, Shreveport, LA 71103 USA; 5 Benchmark Physical Therapy, Upstream Rehabilitation, Knoxville, TN 37920 USA; 6 GMERS Medical College, Gotri, Vadodara, Gujarat 390021 India; 7 Pramukhswami Medical College, Karamsad, Gujarat 388325 India; 8 Department of Neurosurgery, Siloam Hospital Lippo Village, Tangerang, Banten, Indonesia; 9 Kathmandu Medical College and Teaching Hospital, Kathmandu, 44600 Nepal; 10 Department of Pharmacology, Parul Institute of Medical Science and Research, Waghodia, Gujarat 291760 India; 11 Department of Anesthesiology and Interventional Pain, Louisiana State University Health Sciences Center at Shreveport, Shreveport, LA 71103 USA; 12 Louisiana Addiction Research Center, Shreveport, LA 71103 USA

**Keywords:** Neuropathic pain, Chronic pain, Nociceptive pain, Platelet-rich plasma, Neuromodulation, Nanomedicine, Cannabinoids, Stem cells, Gene therapy

## Abstract

**Purpose of Review:**

This manuscript summarizes novel clinical and interventional approaches in the management of chronic, nociceptive, and neuropathic pain.

**Recent Findings:**

Pain can be defined as a feeling of physical or emotional distress caused by an external stimulus. Pain can be grouped into distinct types according to characteristics including neuropathic pain, which is a pain caused by disease or lesion in the sensory nervous system; nociceptive pain, which is pain that can be sharp, aching, or throbbing and is caused by injury to bodily tissues; and chronic pain, which is long lasting or persisting beyond 6 months. With improved understanding of different signaling systems for pain in recent years, there has been an upscale of methods of analgesia to counteract these pathological processes. Novel treatment methods such as use of cannabinoids, stem cells, gene therapy, nanoparticles, monoclonal antibodies, and platelet-rich plasma have played a significant role in improved strategies for therapeutic interventions.

**Summary:**

Although many management options appear to be promising, extensive additional clinical research is warranted to determine best practice strategies in the future for clinicians.

## Introduction

Pain is a pervasive and incapacitating symptom that can significantly affect quality of life. According to the World Health Organization (WHO), up to 20% of adults globally experience pain, which is a significant public health issue [1••]. Complex mechanisms underlying pain pose significant management challenges. Despite advancements in pain management, recently developed novel and powerful techniques have evolving appreciation in treatment strategies. The present investigation, therefore, sought to explore treatment options for specific types of pain, including chronic pain, defined as persistent or recurrent discomfort lasting longer than 6 months, neuropathic pain caused by nervous system dysfunction or impairment, and nociceptive pain resulting from tissue damage or inflammation [2, 3]. By examining the latest advances in pain management, this review sought to provide insights into new and effective methods for alleviating pain.

The goal of pain treatment is to reduce suffering and regain function using a comprehensive strategy that combines pharmacological, nonpharmaceutical, and interventional approaches. It is possible to utilize pharmaceutical therapies, such as opioids and NSAIDs; non-pharmacological alternatives, including cognitive-behavioral and physical therapy; and interventional procedures, such as nerve blocks [4, 5]. However, limitations include low success rates and potential negative impacts, such as abuse, dependence development, and risks associated with invasive procedures.

Over the past few years, an increasing effort has emerged with pioneering and inventive approaches to alleviate pain that can surmount inadequacies and limitations of traditional means and furnish superior options for easing discomfort. These modalities include nanomedicine, gene therapy, stem cell therapy, axon therapy, cannabinoid treatment, and other advanced technologies.

Nanomedicine for pain management has seen recent developments in nanoscale drug delivery systems for targeted relief and novel therapies such as tissue engineering [6••, 7]. Nanofiber scaffolds support stem cell growth and promote tissue regeneration and repair. Current developments in gene therapy for pain treatment include use of adeno-associated viruses (AAV) to transfer pain-inhibiting genes into cells, RNA interference (RNAi) to mute pain-inducing genes, and gene editing tools, such as CRISPR-Cas9, to change or eliminate pain-signaling genes [8, 9, 10••]. Stem cell therapy utilizes mesenchymal stem cells (MSCs) and induced pluripotent stem cells (iPSCs), which possess anti-inflammatory and pain-relieving properties [11, 12]. They effectively reduce pain and inflammation while promoting tissue repair. New developments in cannabinoid therapy include cannabidiol (CBD), a naturally occurring substance with anti-inflammatory and pain-relieving properties [13]. Synthetic cannabinoids, such as dronabinol and nabilone, have been approved for chemotherapy-induced nausea and vomiting and are being researched for their potential to treat pain [14••]. Noninvasive pain management techniques such as virtual reality therapy and transcutaneous electrical nerve stimulation are also gaining popularity. Focusing on potential advantages and difficulties, this review provides an overview of the most innovative pain-relieving methods that are currently available.

## Methods

In the present investigation, we conducted a detailed literature analysis of the different methods available for the treatment of neuropathic, chronic, and nociceptive pain. The specific aim of our review was to evaluate available knowledge regarding the importance of different methods of pain management by elaborating history, pathophysiology, mechanism, and efficacy of each method. This literature review was conducted using the PubMed database by searching for the most relevant papers that emerged using keywords and topics such as nano therapy, stem cell therapy, and significance in pain management, axon therapy, use of cannabinoids in pain, newer techniques and gene therapy, and specific nerve root targets for pain management. With numerous results identified, we selected the most relevant to our search criteria. In this regard, to be more precise in completing our research, we used other sources such as clinicaltrials.gov and Cochrane to obtain information related to novel drugs, targets, and clinical trials.

## Discussion

### Nanomedicine

Nanomedicine is the use of nanocarriers, such as nanoparticles, to provide transportation for drugs to places in the human body they would not normally be able to go. Currently, the main use of nanoparticles in medicine is organic materials such as liposomes [15]. Nanomedicine has exhibited improved efficacy of poorly soluble drugs with smaller doses due to the particle’s ability to increase the bioavailability of a drug [16]. These drug delivery systems have specific parameters they use that are designed to optimize circulation time of a drug, as well as increased specificity for certain tissues/organs [17••]. Additionally, nanomedicine demonstrates the potential ability to safely deliver therapeutic doses of normally toxic substances with minimal local or systemic damage [18].

Emerging research has demonstrated the potential of nanoparticles for the treatment of neuropathic pain. Esketamine, a non-opioid analgesic, has been used to manage refractory neuropathic pain. However, a notable drawback of esketamine is its relatively brief half-life, which necessitates repetitive administration to achieve optimal efficacy [19]. A recent study from 2023 in mouse models has demonstrated the enhancement of delivery of esketamine using a nanoparticle-hydrogel delivery system (NHDS). In this study, we utilized a novel formulation known as a nanostructured hydrogel drug system (NHDS). NHDS involves encapsulation of esketamine within poly(lactic-co-glycolic acid) (PLGA) nanoparticles, which are subsequently embedded in a hydrogel matrix. The esketamine-nanoparticle system was injected into the nerve roots of mice 1–7 days after spinal nerve ligation (SNL) to stimulate neuropathic pain. SNL mice treated with the esketamine-nanoparticle system had a significantly enhanced analgesic effect compared to SNL mice treated solely with esketamine [20]. This innovative approach aims to enhance the delivery of esketamine for optimal therapeutic outcomes.

nZnO is a zinc oxide nanoparticle recently investigated for its potential role in pain management owing to its ability to release zinc ions. Zinc acts as a non-competitive inhibitor of the N-methyl-D-aspartate (NMDA) receptor, a type of glutamate receptor known for its involvement in hyperalgesia, neuropathic pain, and impaired functioning of opioid receptors. Therefore, nZnO may be used to increase zinc bioavailability at certain sites, which would then decrease the ability of glutamate to act on NMDA receptors at those sites. This would then produce an analgesic effect [21]. Additionally, nZnO is thought to play a role in enhancing opioid receptor activity which suggests that when given together along with morphine would increase its anti-nociceptive effect. Nanoparticles carrying other ionic compounds such as magnesium oxide (MgO), manganese dioxide (MnO_2_), and magnetite (Fe_3_O_4_) are also being investigated due to their ability to release pain with mechanisms similar to those of nZnO [22].

While the role and use of nanoparticles in medicine is still evolving, more research is needed to establish their clinical effectiveness. However, its potential is highly promising. Nanoparticles possess unique properties that enable them to serve as carriers for enhanced drug delivery. This characteristic opens up possibilities not only for pain management but also for addressing various chronic health conditions.

### Gene Therapy

Gene therapy was first introduced as a potential strategy for treating recessive-inherited disorders. This concept centered on the idea of introducing a functional copy of a mutated gene to restore normal cellular function. With a few exceptions, gene therapy has shown success in various applications. However, its primary emphasis has shifted towards targeting acquired diseases, aiming to modify their underlying processes [23••].

Gene therapy can be classified into two main types: somatic and germline. Somatic gene therapy aims to treat patients directly without altering the genetic makeup of their offspring. This involves the introduction of a functional gene into specific cells, thereby correcting the underlying genetic defects. However, it is important to note that even if somatic gene therapy successfully modifies a patient’s genes to address a particular condition, the risk of passing on the ailment to future generations remains. This form of gene therapy is practiced in genetic laboratories worldwide.

In contrast, germline gene therapy involves inserting exogenous genes into cells involved in producing sperm or fertilized eggs. As a result, any genetic modification to these cells can be inherited by the offspring. Although germline gene therapy has the potential to prevent hereditary diseases, it is a highly controversial area of research owing to technical and ethical concerns [24]. Currently, limited research has been conducted in this field.

In addition to these two types, various methods have been employed for gene therapy. These include the direct injection of DNA, liposome-mediated DNA transfer, calcium phosphate transfection, electroporation, the use of retrovirus vectors, site-directed recombination, artificial chromosomes, other viral vectors, targeted gene transfer via receptors, and gene activation with associated activities [25••]. Each method offers unique advantages and considerations depending on the specific goals of the gene therapy intervention.

Studies have shown the use of gene therapy to inhibit nociceptive transmission at the supraspinal level [26••]. Previous experimental studies on gene delivery in the brain have mainly focused on the medulla oblongata, a region responsible for pain control, which presents challenges owing to its proximity to vital organ systems like the pulmonary and cardiovascular. To address this, it may be advantageous to redirect research efforts towards more accessible locations, especially in the context of prevalent conditions associated with chronic pain such as complex regional pain syndrome or fibromyalgia. Targeting various points along the pain pathway is feasible with gene therapy, but particular attention is given to primary afferent neurons (PANs) and their synapses, as well as second-order neurons inside the dorsal horn, owing to their significant involvement in the pathophysiological development of chronic pain [27].

The concept of gene therapy for pain is solely based on the way genes are delivered that encodes the proteins that block the nociceptive receptors or would interfere with the nociceptive molecule inside the functional unit of the brain, so-called neurons. Introduction of certain genes into neurons, where they produce particular proteins, is the basis of gene therapy for persistent neuropathic pain. These proteins act on the ionic channels and receptors implicated in the development of pain due to neuropathy [28••]. This is achieved either by viral method, which is called transfection, or non-viral vector methods known as transduction.

To deliver analgesic genes in the nervous system, three gene therapy approaches have been used: cell-based therapies, which typically involve transplanting mutated cells into the subarachnoid space [29••, 30, 31]; the use of plasmids and oligonucleotides, which are occasionally enclosed in the liposomes to facilitate entry into cells [32–34]; and the use of certain viral-based vectors. The most popular method for delivering genes to the nervous system is viral vector gene therapy, which makes use of viruses’ innate capacity to transmit infection for their genes to be expressed by host cells [35, 36].

It has been previously demonstrated that HSV-based vector-induced transfer of the ENK gene to rodent’s dorsal root ganglia in the lumbar region through footpad inoculation lowers C-fiber-evoked pain responses in reflex to immediate inflammatory stimuli, such as a capsaicin injection [37] or formalin [27], and attune the increased sensitivity to pain related to chronic inflammation [38, 39]. In addition, implementation of the rat vibrissal pad has been shown to reduce neuropathic pain related to trigeminal neuralgia [40]. The method of footpad inoculation has also been shown to abridge nociceptive behavior in mouse or rat models of chronic neuropathic pain that also includes pain due to diabetic neuropathy and spinal nerve ligation [41••, 42••]. Since then, other researchers have demonstrated that visceral pain models of cystitis and pancreatitis have elicited analgesia when ENK-expressing HSV vectors are injected directly into the bladder or pancreas and have their expanded findings to primate models [43–46]. The combination of all these researches was the projections towards the first ever human gene experiment for the management of pain.

In a partial sciatic nerve damage model of neuropathic pain in rodents, McMahon et al. showed that the glial cell line–derived neurotrophic factor (GDNF) direct intrathecal infusion lowered pain and its related behaviors along with ectopic nerve discharges [47]. Multiple studies have demonstrated that administration of the recombinant p75 soluble TNFR (sTNFR) peptide (etanercept) intrathecally depleted mechanical allodynia in a mouse model of neuropathic pain, while counteracting antibodies aimed against the p55 TNF receptor (TNFR) reduced hyperalgesia related to the heat and mechanical allodynia [48–50]. The Watkins group has shown in rat tests that intrathecal mode of injection of adenovirus plus the adeno-associated virus or simple plasmids engineered to produce IL-10 results in the liberation of IL-10 into the cerebrospinal fluid. In prototype of neuropathic pain generated by either nerve compression or injecting allogenic chemicals along the sheath of the nerve, the anti-inflammatory cytokine can either reverse existing neuropathic pain or prevent it from occurring [51–53].

Non-viral vectors are also vital for gene therapy, but there is not much evidence supporting their role in pain management. The efficiency and specificity of non-viral vectors in comparison to viral vectors are lower. Besides the non-viral vector method, there are different ways to deliver gene threads, namely, by polymers that are either biodegradable or non-biodegradable, lipids that are conventional, gemini surfactants, lipidoids, helper lipids, peptides (peptic nucleic acid, polypeptides, functional peptides), inorganic materials such as mesoporous silica nanoparticles, gold nanoparticles, magnetic materials, carbon nanotubes and graphene, quantum dots, and hybrid systems (inorganic-organic, modified PEI, inorganic lipid, and peptide bond).

Gene therapy has its own set of limitations such as dosing, safety, and efficacy, apart from other drawbacks such as the ethical issues of gene therapy, as it will change not only the physical but also the expressive side of a protein to attain a positive result in the process of dealing with chronic pain. Despite the immense potential of gene therapy as a medical treatment, progress in the development of efficient clinical procedures has been gradual. The development of reliable and effective gene delivery devices is an issue. Although gene therapy works well for single-gene problems, additional studies are needed for multiple gene disorders. Although viruses are effective gene carriers, they have certain drawbacks. To improve target specificity and lessen injury to nearby healthy tissues, further research must be done to create novel, efficient gene carriers [25].

### Stem Cell Therapy

Stem cells possess the extraordinary ability to differentiate into various cell types within the human body. They play pivotal roles in embryonic development and contribute significantly to organ formation. In adulthood, they continue to perform vital functions by aiding organ repair and tissue regeneration. Stem cells can be classified into diverse types, including adult, embryonic, pluripotent, and multipotent. Furthermore, their differentiation potential allows them to be categorized based on their specialization into specific cell types. During the pilot stages of fertilized ovum division, totipotent cells emerge, possessing a remarkable capacity to differentiate into any type of cell, thus enabling the creation of complete organs. On the other hand, pluripotent cells, such as embryonic stem cells and the induced pluripotent stem cells (iPSCs), demonstrate the caliber to adapt into various cell types but lack the capacity to develop into entire organs [12].

To enhance our understanding of this field, it is important to recognize that several types of stem cells originate from distinct sources. The innate cell mass of the blastocysts serves as a genesis of embryonic stem cells, which have a remarkable capacity to differentiate into the three germ layers. Adult stem cells can be classified based on their tissue of origin, including those derived from the placenta and umbilical cord, hematopoietic stem cells, mesenchymal cells derived from the bone marrow, and mesenchymal cells (MSCs) from adipose tissue. These adult stem cells play a crucial part in the renewal and repair of injured tissues or organs and are distributed throughout various tissues in the body [54]. Stem cell therapy may effectively address neuropathic pain associated with conditions, such as sciatic nerve injury, diabetes-induced neuropathies, and spinal cord injury [55••]. By utilizing stem cells to treat neuropathic pain, the effects of refractory pain can be modulated and reversed. This treatment strategy makes use of stem cell capacity to generate neurotrophic factors as a biological origin for regenerating damaged brain cells [56].

Human MSCs have emerged as the preferred cell type for the treatment of lower back pain. Similar to MSCs obtained from the bone marrow, the nucleus pulposus also contains MSCs, and co-culturing them with nucleus pulposus cells enhances both the proliferation of nucleus pulposus cells and the differentiation of MSCs towards the chondrogenic lineage [57••]. Vadivelu et al. conducted three animal model studies exploring the usage of stem cells in the treatment of diabetic neuropathy, with stem cells being administered through intramuscular injections in the posterior portion of the leg. MSCs were selected because of their capacity to differentiate into various cell types and their ability to secrete cytokines. Furthermore, recent research has suggested that MSCs may promote neurotrophic factors, which are often depleted in diabetic neuropathy, proving the use of these types of stem cells beneficial. One study utilized marrow mononuclear cells because of their accessibility, and improvements were observed between 2 and 15 weeks after the injury. These investigations collectively demonstrate the efficacy of this unique approach in treating neuropathic-related pain [12, 55].

Mesenchymal stem cells (MSCs) have emerged as crucial contributors to nerve healing and regeneration, and their ability to release cytokines suggests a potential anti-inflammatory effect that can combat pathological inflammation linked to neuropathic pain [58–60]. This concept had been scrutinized in animal models of trigeminal neuropathy, diabetic neuropathy, and dorsal paw neuropathy, yielding encouraging results marked by the significant alleviation of neuropathic pain symptoms [61, 62, 63••]. These findings underscore the essential role of stem cells in addressing chronic, neuropathic, and nociceptive pain.

The choice between allogeneic and autologous stem cell types needs to be carefully considered for appropriate use. Although only a few human trials are available, they effectively demonstrate the beneficial aspects of stem cell applications. For instance, in a case study, a patient with a crush fracture in the L1 vertebral body and partial spinal cord injury at the level of T12-L1 region received multiple doses of allogeneic MSCs via intrathecal and intravenous administration, resulting in a significant reduction in neuropathic pain [64]. Early transplantation of MSCs is hypothesized to enhance functional recovery through various mechanisms, including the reduction of gliosis, suppression of inflammatory cytokine production, promotion of spinal cord revascularization via angiogenic effects, and stimulation of bioactive molecules and growth factor production. In a recent study, adipose-derived MSCs were used to treat neuropathic facial pain in eight patients who failed pharmacotherapy. These patients received perineural injections of stem cells into the damaged portion of the trigeminal nerve, leading to a significant decrease in the mean pain score from 7.5 to 4.3 out of 10 after 6 months. Most patients (seven out of nine) responded positively to the treatment, with five of them experiencing reduced gabapentin requirements. Notably, no significant side effects such as infections were observed [65].

The morphology, differentiation, viability, and migratory capabilities of mesenchymal stem cells (MSCs) are influenced by the duration and intensity of their growth and culture. During cultivation and passage, MSCs undergo phenotypic changes in size, morphology, and cell surface marker expression, while simultaneously experiencing a gradual decline in their proliferative and functional differentiation potential [66, 67]. Additionally, the proliferation and differentiation abilities of cytokines are also affected [67]. Determining the appropriate dosage of stem cells poses a challenge. Nonetheless, further investigation of the types of stem cells, dosages, safety considerations, and implantation rates is necessary for effective therapeutic applications. In a mouse model, the repeated administration of neuronal or adipose stem cells demonstrates a dose-dependent analgesic effect [68••].

Although stem cell therapy represents a promising and effective approach for managing chronic neuropathic and nociceptive pain, it has certain limitations that warrant attention. These include concerns regarding the potential development of teratomas, the optimal dosage and timing of stem cell therapy, and a comprehensive evaluation of the benefits and drawbacks associated with its use. Further research studies and clinical trials that meet rigorous standards can provide a solid foundation for assessing the impact of stem cell therapy, not only in various diseases but also in pain modulation and management.

### Axon Therapy or Transcutaneous Magnetic Stimulation (tMS)

Peripheral nerve injury commonly leads to development of neuromas or nerve entrapment, resulting in persistent neuropathic pain. This pain condition is characterized by heightened activity occurring either at the site of injury or at the dorsal root ganglion [69]. Axon therapy, also known as transcutaneous magnetic stimulation (tMS), is derived from transcranial magnetic stimulation (TMS), with tMS being a localized and targeted version of TMS [70]. Unlike TMS, tMS utilizes a device that generates small electrical currents around neuromas, eliminating the need for anesthetics. By passing a dynamic magnetic flux through the skin, this device effectively reaches the first few centimeters without any attenuation. Although the mechanism of action is not fully understood, TMS has been demonstrated to facilitate nerve repair and regeneration. In particular, low-frequency TMS has shown inhibitory effects on neurons and may serve as a viable treatment option for patients with neuromas [70].

The TMS method was developed in the 1950s, but it was not until Barker and colleagues [71] established noninvasive magnetic stimulation in 1985 that the application of this therapy was broadened. Axon therapy, employing transcranial magnetic stimulation (TMS), shows promise in alleviating chronic or long-term pain subject to crucial criteria. First, it induces plastic changes in the central nervous system (CNS) through nociceptive inputs, thereby contributing to the maintenance of pain experience. Second, TMS must have the ability to elicit plastic changes within the CNS. Finally, the plastic changes induced by TMS should effectively counteract the alterations induced by nociception. These changes are noticeable at both cellular and physiologic levels [72].

Effective management of pain with the prospect of magnetic stimulation may require an integration of appropriate targets that suit the specific conditions and symptoms along with different stimulation types. For example, a former study demonstrated that the priming paradigm, involving the initial administration of iTBS (theta burst stimulation) to M1, can enhance the analgesic effects of high-frequency TMS [73]. As fascinating as it sounds, there is not enough evidence supporting it and more elaborate research is necessary.

Studies have shown that, in healthy persons, increased frequency rTMS applied to the primary motor cortex (M1) induces rapid modifications and modulation within the sensorimotor networks [74]. Furthermore, empirical evidence indicates that high-frequency rTMS can directly influence sensory thresholds for both cool and humid temperature sensations. This suggests its potential utility in alleviating symptoms experienced by individuals with chronic pain.

A recent systematic review showed that high-frequency repetitive transcranial magnetic stimulation (rTMS) of the left primary motor cortex (M1) is effective in depleting pain related to the fibromyalgia syndrome. Importantly, the analgesic effects of this stimulation lasted beyond the duration of actual stimulation [75]. In contrast, a Cochrane systematic review conducted recently examined the use of rTMS for chronic pain management. This review revealed that collaborative doses of rTMS do not consistently demonstrate merits across trials. However, one dose of rTMS has short-term positive effects in individuals with long-term pain [76, 77].

A study from 2014 investigated possible benefits for patients who have resistant neuropathic pain for treatment with tMS. In this study, 5 patients had neuropathic pain that was resistant to previous therapies, including non-steroidal anti-inflammatory drugs (NSAIDs), corticosteroids, lidocaine, vicodin, and gabapentin. All 5 patients were subjected to 3–4 tMS sessions at a frequency of 0.5 Hz. The average pain reduction in the numerical rating pain scale (NRS) was 84%, with 3 out of the 5 patients reporting a complete loss of neuropathic pain [70].

A recent study conducted in 2021 investigated the effectiveness of tMS in the treatment of painful diabetic neuropathy and found comparable results. In this 10 participant study, 9 of them completed the entire study, in which an average of 1.22 ± 1.79-point decrease in pain was reported. This correlates to about a 78% improvement from baseline, with five participants reporting a 100% improvement. Pain reductions were significant up to 7-day post-treatment (*P* = 0.0295) [78]. While these studies showed very promising results in patients who were responsive to treatment, studies with higher patient participation and longitudinal studies are needed before determining clinical efficacy and implementation.

rTMS has demonstrated significant analgesic effects in patients with long-lasting refractory pain syndromes, including post-stroke pain involving the thalamus or brainstem, spinal cord injury pain, nerve root/brachial plexus avulsion pain, trigeminal neuropathy pain, migraine, and fibromyalgia. However, there is currently limited understanding of the effectiveness of rTMS, specifically for different pain conditions. The brief period of reduced sensation to pain provided by transcranial stimulation, which is a drawback of rTMS, presents challenges in determining its effectiveness in individuals with chronic pain syndrome [79].

### Cannabinoid in Pain

Cannabinoids are a class of chemical compounds that naturally occur in *Cannabis* plants. Historically, cannabis has been used as a recreational drug owing to its psychoactive properties. However, emerging research suggests that cannabinoids may have the ability to treat pain. These compounds primarily interact with two types of receptors: CB1 and CB2. The former receptor is predominantly found in the central nervous system (CNS), while the CB2 receptors are primarily present in immune system organs like the spleen and tonsils [80, 81]. The abundance of CB1 receptors in the CNS suggests their involvement in the addictive and psychoactive properties associated with cannabinoids [82].

In contrast, the precise method by which CB2 receptors contribute to the reduction of neuropathic pain remains unclear. One proposed theory suggests that CB2 receptors achieve this effect through inhibition of T-cell receptor (TCR) signaling. CB2 receptor is a member of the Gi/o G-protein-coupled receptor family, which subsequently inhibits the activity of adenylyl cyclase, an enzyme responsible for converting adenosine triphosphate (ATP) into cyclic adenosine monophosphate (cAMP). The resulting decrease in cAMP levels affects a signaling pathway that inhibits TCR signaling, thereby reducing immune system activation and inflammation, and ultimately leading to pain reduction [83, 84].

Damage to the nervous system is linked to a significant increase in the expression of CB2 receptors in the dorsal root ganglion (DRG), suggesting that targeting the CB2 receptor could be a potential approach for treating neuropathic pain [85]. (-)-Delta9-trans-tetrahydrocannabinol (THC), the primary cannabinoid found in cannabis, acts as a partial agonist of both CB1 and CB2 receptors and is associated with the psychoactive and pain-relieving properties of the plant. A recent systematic review and meta-analysis has indicated that interventions involving THC result in statistically significant improvements in pain reduction among patients with neuropathic pain compared to placebo [86]. However, further long-term studies are necessary to establish clear clinical guidelines and recommendations in this regard.

While treating a patient with neuropathic pain, the THC dosage is vital. A recent randomized placebo-controlled trial conducted in 2020 investigated the effectiveness of inhaled THC in pain management. The study revealed that administering an inhaled THC dose of 1.0 mg resulted in a significant reduction in patient scores on the visual analog pain scale (VAS), with a maximum mean decrease of 2.95 points. In comparison, patients who received a lower dose of 0.5 mg THC experienced a decrease of 1.95 points on the VAS [87••].

Although many studies have indicated the possible benefits of THC for chronic and neuropathic pain, additional long-term studies are needed to achieve a clear clinical reference and to determine if the analgesic benefits outweigh the possible adverse effects. The same clinical trial mentioned in the last paragraph saw that many of the participants reported adverse effects such as a drug high, cough, and weakness [87••]. Other reported side effects with THC include, but are not limited to, cognitive adverse reactions, such as cognitive impairment and altered mental status [88].

### Recent Advancements

#### Neuromodulation

Neuropathic pain is a widespread health issue affecting approximately 17% of the overall population [89••]. The primary treatment options for this type of pain involve the use of antidepressants and anticonvulsants. Tricyclic antidepressants (TCAs) and serotonin-noradrenaline reuptake inhibitors (SSRIs) are commonly prescribed as antidepressant medications, whereas gabapentin and pregabalin are frequently used as anticonvulsant drugs. It is noteworthy that gabapentin and pregabalin share a similar mechanism of action [90].

Neuromodulation is an emerging field of biotechnology that has gained immense momentum. It offers a non-pharmacological approach for treating neuropathic pain by modulating the central or peripheral nervous system [91]. Various techniques are employed in neuromodulation, all of which share the common objective of using electrical, magnetic, or optogenetic energy to target specific areas of the nervous system. In doing so, they aim to suppress disease states and restore the system to a healthy state. Neuromodulation shows promise as an alternative treatment for chronic lower back pain and may potentially reduce reliance on opioids among patients [92••]. Although the underlying neurophysiological mechanisms of neuromodulation and its ability to alleviate neuropathic pain are not yet fully understood, they hold exciting potential.

Further research is necessary to establish a clear clinical relevance and enhance our understanding of this promising approach.

#### Platelet-Rich Plasma

Platelet-rich plasma (PRP) therapy is a treatment modality that involves injecting a concentrated solution of platelets, which subsequently releases growth factors to initiate the healing process in connective tissues. In recent times, PRP therapy has gained popularity as an approach to managing chronic pain, particularly back pain, owing to its favorable side effect profile and success rates. Conservative treatment options, such as physical therapy, non-steroidal anti-inflammatory drugs (NSAIDs), and glucocorticoids, are initially employed for chronic back pain, with surgery considered only when these interventions prove ineffective.

However, PRP therapy is an alternative because of its ability to enhance the body’s natural healing mechanisms [93].

In addition to a role in hemostasis, platelets serve several other essential functions in the body. For instance, they facilitate the recruitment of white blood cells to the site of tissue injury, aiding in the prevention of infection of damaged cells. Moreover, platelets contain platelet-derived growth factor (PDGF), a growth factor that stimulates stem cell formation. This has rendered platelets appealing for the treatment of osteoarthritis (OA). Prolotherapy (PRL) is often recommended as a viable therapeutic option in cases of persistent musculoskeletal and painful joint diseases such as knee OA [94].

In a comprehensive systematic review conducted in 2017, comprising 14 randomized controlled trials (RCTs), it was found that intra-articular platelet-rich plasma (PRP) injections were effective in significantly reducing pain among individuals with knee osteoarthritis (OA) when compared to alternative intra-articular injections, such as hyaluronic acid, ozone, saline, and corticosteroids ^94^. Nonetheless, it should be noted that one limitation of PRP therapy is its potential inability to fully alleviate acute pain, thus necessitating additional supplementation with painkillers during the initial stages of treatment [95].

Platelet-rich plasma (PRP) therapy is gaining popularity as an effective treatment for discogenic back pain. It offers a safe and successful therapeutic option, with a reported success rate of 71.2% and high patient satisfaction in long-term outcomes [96••]. M. Hussein and T. Hussein conducted research demonstrating that the injection of PRP into atrophied lumbar multifidus muscles is a secure and efficient method for managing persistent low back pain and related impairments. However, it is important to note that this trial did not include a comparison group [97].

The outcomes of PRP therapy can be influenced by factors such as variations in the preparation and composition of the treatment and the specific anatomical and medical conditions of individuals. Navani et al. [98] demonstrated the benefits of locally administered PRP injections in promoting tissue recovery in painful musculoskeletal conditions. Moreover, several studies have highlighted the potential of PRP as an alternative treatment option for diabetic neuropathy (DBN), showing significant pain reduction in DBN patients [99–101].

Decisions about the use of PRP therapy, which is more expensive than PRL because it requires centrifuge equipment and specialized supplies, must be made carefully [100]. The other set of limitations is pain and bleeding at the site of injection, which itself can be a setback in the use of PRP for the management of pain. In addition, adequate dosage and efficiency should be determined for its consideration as a line of treatment for pain. The future of PRP can be determined in various upcoming studies, and it can prove to be one of the most vital management options.

#### Monoclonal Antibodies

Monoclonal antibodies (mAbs) are antibodies that bind specifically to the same epitope of the same antigen, disrupting the intended pathway stimulated by the antigen. Currently, mAbs are extensively utilized for treating inflammatory autoimmune conditions, such as rheumatoid arthritis, psoriatic arthritis, and ankylosing spondylitis. However, recent research has explored the potential of mAbs as a therapy for chronic pain by targeting specific ligands involved in neurogenic inflammation and pain transmission [102]. Notably, proteins such as high-mobility group box-1 (HMGB1) and nerve growth factor (NGF) have emerged as important targets for mAbs in chronic pain treatment because of their involvement in the release of pro-inflammatory molecules and stimulation of pain [103]. Recent studies have demonstrated promising outcomes in using mAbs to alleviate chronic pain, although several adverse effects, including paresthesia, peripheral edema, and arthralgias, have also been reported in these studies.

The primary mechanism of NGF-induced pain signaling involves tropomyosin receptor kinase A (TrkA), which is its main target. By forming a highly stable complex with NGF, TrkA establishes a durable interaction that persists for more than 100 h. This interaction enhances the autophosphorylation of the TrkA intracellular domain, facilitating the activation of the mitogen- activated protein kinase (MAPK) pathway, specifically in nociceptive terminals. Subsequently, this activation sets in motion a series of downstream pain-signaling cascades [104, 105••, 106]. Furthermore, NGF exerts its influence by promoting the release of pain mediators such as substance P. These mediators play a role in intensifying the pain response. NGF contributes to the sensitization of nociceptive neurons, making them more responsive to painful stimuli. This sensitization occurs through the upregulation of ion channels and receptors present on primary afferent nerve fibers [105••, 106]. Notably, NGF may sensitize nociceptors in cases of neuropathic pain, leading to a condition known as hyperalgesia [107].

Numerous studies have demonstrated the effectiveness of TNF-α, a cytokine with diverse effects on inflammation, in alleviating inflammation-related symptoms and signs associated with diseases, such as rheumatoid arthritis (RA) and complex regional pain syndrome. TNF-α also plays a complex role in both the central and peripheral pathways involved in pain transmission. To specifically target TNF-alpha, monoclonal antibodies (mAbs) such as adalimumab, etanercept, and infliximab have been developed and extensively investigated in chronic autoimmune inflammatory disorders characterized by prominent pain symptoms, notably rheumatoid arthritis. Differentiating the anti-inflammatory properties of TNF-α inhibitors from their effects on pain-specific pathways presents significant challenges [108–111].

Researchers have explored the potential of mAb TNF-alpha antagonists in treating conditions like sciatica, chronic low back pain (CLBP) with or without radiculopathy, and ankylosing spondylitis [112••].

Before broad clinical application of monoclonal antibodies (mAbs) for chronic pain can be considered, several challenges must be overcome. To begin with, excluding tanezumab, the findings of specific clinical studies have not been particularly noteworthy [113]. Secondly, it is unclear how many mAbs that are being developed for these purposes will be tolerated and safe. Third, the substantial cost remains a significant practical consideration when considering the utilization of monoclonal antibodies (mAbs) for chronic pain, potentially limiting their widespread use. Based on these restrictions, some have questioned whether mAbs would successfully treat chronic pain [113].

## Conclusion

This narrative review effectively integrates all essential approaches to treat chronic, nociceptive, and neuropathic pain incorporating the latest therapeutic options. Considering the increasing prevalence of chronic pain in recent years, the adoption of these treatment modalities has become quintessential. Even though we have several other alternatives among the available management options, we need to dig deeper via future clinical research to fully fill the demands of this evolving issues in pain medicine, with fewer side effects and appropriate safety results.

## References

[1] Rice ASC, Smith BH, Blyth FM (2016). Pain and the global burden of disease. Pain.

[2] Kumar K, Elavarasi P, David CM (2016). Definition of pain and classification of pain disorders. J Adv Clin Res Insights.

[3] Swieboda P, Filip R, Prystupa A, Drozd M. Assessment of pain: types, mechanism and treatment. Ann Agric Environ Med. 2013;Spec no. 1:2–7. 25000833

[4] Boldt I, Eriks-Hoogland I, Brinkhof MW, de Bie R, Joggi D, von Elm E. Non-pharmacological interventions for chronic pain in people with spinal cord injury. Cochrane Database Syst Rev. 2014;(11):CD009177. Published 2014 Nov 28. 10.1002/14651858.CD009177.pub2. 10.1002/14651858.CD009177.pub2 PMC11329868 25432061

[5] Reid MC, Eccleston C, Pillemer K (2015). Management of chronic pain in older adults. BMJ.

[6] Bhandari R, Sharma A, Kuhad A (2021). Novel nanotechnological approaches for targeting dorsal root ganglion (DRG) in mitigating diabetic neuropathic pain (DNP). Front Endocrinol (Lausanne).

[7] Xiao L, Cui J, Sun Z (2022). Therapeutic potential of nanotechnology-based approaches in osteoarthritis. Front Pharmacol.

[8] Hu C, He M, Xu Q (2021). Advances with non-coding RNAs in neuropathic pain. Front Neurosci.

[9] Kim D, Kim KR, Kwon Y (2020). AAV-mediated combination gene therapy for neuropathic pain: GAD65, GDNF, and IL-10. Mol Ther Methods Clin Dev.

[10] Karimian A, Azizian K, Parsian H (2019). CRISPR/Cas9 technology as a potent molecular tool for gene therapy. J Cell Physiol.

[11] Joshi HP, Jo HJ, Kim YH, An SB, Park CK, Han I. Stem Cell Therapy for Modulating Neuroinflammation in Neuropathic Pain. Int J Mol Sci. 2021;22(9):4853. Published 2021 May 3. 10.3390/ijms22094853. 10.3390/ijms22094853 PMC8124149 34063721

[12] Padda J, Khalid K, Zubair U (2021). Stem cell therapy and its significance in pain management. Cureus.

[13] Alkislar I, Miller AR, Hohmann AG (2021). Inhaled cannabis suppresses chemotherapy- induced neuropathic nociception by decoupling the raphe nucleus: a functional imaging study in rats. Biol Psychiatry Cogn Neurosci Neuroimaging.

[14] Atigari DV, Paton KF, Uprety R (2021). The mixed kappa and delta opioid receptor agonist, MP1104, attenuates chemotherapy-induced neuropathic pain. Neuropharmacology.

[15] Chen J, Jin T, Zhang H (2020). Nanotechnology in chronic pain relief. Front Bioeng Biotechnol.

[16] Jia L (2005). Nanoparticle formulation increases oral bioavailability of poorly soluble drugs: approaches experimental evidences and theory. Curr Nanosci.

[17] •• Bhansali D, Teng SL, Lee CS, Schmidt BL, Bunnett NW, Leong KW. Nanotechnology for Pain Management: Current and Future Therapeutic Interventions. Nano Today. 2021;39:101223. 10.1016/j.nantod.2021.101223. **Excellent review on advances in nanoparticle-based drug delivery to reduce side effects, gene therapy to tackle the source of pain, and nanomaterial-based scavenging to proactively mediate pain signaling.** 10.1016/j.nantod.2021.101223 PMC8654201 34899962

[18] Zhao C, Liu A, Santamaria CM (2019). Polymer-tetrodotoxin conjugates to induce prolonged duration local anesthesia with minimal toxicity. Nat Commun.

[19] Meissner K, Henthorn TK (2021). How relevant is stereoselectivity to the side-effects of ketamine?. Br J Anaesth.

[20] Zhang H, Zhou P, Jiang Y (2023). Sustained-release esketamine based nanoparticle- hydrogel delivery system for neuropathic pain management. Int J Nanomedicine.

[21] Babaie S, Taghvimi A, Hong JH (2022). Recent advances in pain management based on nanoparticle technologies. J Nanobiotechnology.

[22] Kesmati M, Torabi M (2014). Interaction between analgesic effect of nano and conventional size of zinc oxide and opioidergic system activity in animal model of acute pain. Basic Clin Neurosci.

[23] Glorioso JC, Fink DJ (2009). Gene therapy for pain: introduction to the special issue. Gene Therapy.

[24] Moss JA. Gene therapy review. Radiol Technol. 2014;86(2):155–184. 25391667

[25] Mali S (2013). Delivery systems for gene therapy. Indian J Hum Genet.

[26] Wu CL, Garry MG, Zollo RA (2001). Gene therapy for the management of pain: part I: methods and strategies. Anesthesiology.

[27] Goss JR, Krisky D, Wechuck J (2014). Herpes simplex virus-based nerve targeting gene therapy in pain management. J Pain Res.

[28] Kumar S, Ruchi R, James SR (2011). Gene therapy for chronic neuropathic pain: how does it work and where do we stand today?. Pain Med.

[29] Eaton MJ, Plunkett JA, Martinez MA (1999). Transplants of neuronal cells bioengineered to synthesize GABA alleviate chronic neuropathic pain. Cell Transplant.

[30] Ishii K, Isono M, Inoue R (2000). Attempted gene therapy for intractable pain: dexamethasone-mediated exogenous control of beta-endorphin secretion in genetically modified cells and intrathecal transplantation. Exp Neurol.

[31] Duplan H, Li RY, Vue C (2004). Grafts of immortalized chromaffin cells bio-engineered to improve met-enkephalin release also reduce formalin-evoked c-fos expression in rat spinal cord. Neurosci Lett.

[32] Luo D, Saltzman WM (2000). Synthetic DNA delivery systems. Nat Biotechnol.

[33] Yao MZ, Gu JF, Wang JH (2002). Interleukin-2 gene therapy of chronic neuropathic pain. Neuroscience.

[34] Wu CM, Lin MW, Cheng JT (2004). Regulated, electroporation-mediated delivery of pro- opiomelanocortin gene suppresses chronic constriction injury-induced neuropathic pain in rats. Gene Ther.

[35] Davidson BL, Breakefield XO (2003). Viral vectors for gene delivery to the nervous system. Nat Rev Neurosci.

[36] Kay MA, Glorioso JC, Naldini L (2001). Viral vectors for gene therapy: the art of turning infectious agents into vehicles of therapeutics. Nat Med.

[37] Wilson SP, Yeomans DC, Bender MA (1999). Antihyperalgesic effects of infection with a preproenkephalin-encoding herpes virus. Proc Natl Acad Sci USA.

[38] Yeomans DC, Jones T, Laurito CE (2004). Reversal of ongoing thermal hyperalgesia in mice by a recombinant herpesvirus that encodes human preproenkephalin. Mol Ther.

[39] Braz J, Beaufour C, Coutaux A (2001). Therapeutic efficacy in experimental polyarthritis of viral-driven enkephalin overproduction in sensory neurons. J Neurosci.

[40] Meunier A, Latremoliere A, Mauborgne A (2005). Attenuation of pain-related behavior in a rat model of trigeminal neuropathic pain by viral-driven enkephalin overproduction in trigeminal ganglion neurons. Mol Ther.

[41] Chattopadhyay M, Mata M, Fink DJ (2008). Continuous delta-opioid receptor activation reduces neuronal voltage-gated sodium channel (NaV1.7) levels through activation of protein kinase C in painful diabetic neuropathy. J Neurosci.

[42] Hao S, Mata M, Goins W (2003). Transgene-mediated enkephalin release enhances the effect of morphine and evades tolerance to produce a sustained antiallodynic effect in neuropathic pain. Pain.

[43] Yang H, McNearney TA, Chu R (2008). Enkephalin-encoding herpes simplex virus-1 decreases inflammation and hotplate sensitivity in a chronic pancreatitis model. Mol Pain.

[44] Yokoyama H, Sasaki K, Franks ME (2009). Gene therapy for bladder overactivity and nociception with herpes simplex virus vectors expressing preproenkephalin. Hum Gene Ther.

[45] Lu Y, McNearney TA, Lin W (2007). Treatment of inflamed pancreas with enkephalin encoding HSV-1 recombinant vector reduces inflammatory damage and behavioral sequelae. Mol Ther.

[46] Yeomans DC, Lu Y, Laurito CE (2006). Recombinant herpes vector-mediated analgesia in a primate model of hyperalgesia. Mol Ther.

[47] Bennett DL, Boucher TJ, Armanini MP (2000). The glial cell line-derived neurotrophic factor family receptor components are differentially regulated within sensory neurons after nerve injury. J Neurosci.

[48] Sommer C, Schmidt C, George A (1998). Hyperalgesia in experimental neuropathy is dependent on the TNF receptor 1. Exp Neurol.

[49] Schafers M, Svensson CI, Sommer C (2003). Tumor necrosis factor-alpha induces mechanical allodynia after spinal nerve ligation by activation of p38 MAPK in primary sensory neurons. J Neurosci.

[50] Svensson CI, Schafers M, Jones TL (2005). Spinal blockade of TNF blocks spinal nerve ligation-induced increases in spinal P-p38. Neurosci Lett.

[51] Milligan ED, Langer SJ, Sloane EM (2005). Controlling pathological pain by adenovirally driven spinal production of the anti-inflammatory cytokine, interleukin-10. Eur J Neurosci.

[52] Milligan ED, Sloane EM, Langer SJ (2005). Controlling neuropathic pain by adeno-associated virus driven production of the anti-inflammatory cytokine, interleukin-10. Mol Pain.

[53] Ledeboer A, Sloane EM, Milligan ED (2005). Minocycline attenuates mechanical allodynia and proinflammatory cytokine expression in rat models of pain facilitation. Pain.

[54] Biehl JK, Russell B. Introduction to stem cell therapy. J Cardiovasc Nurs. 2009;24(2):98–105. 10.1097/JCN.0b013e318197a6a5. 10.1097/JCN.0b013e318197a6a5 PMC4104807 19242274

[55] Vadivelu S, Willsey M, Curry DJ (2013). Potential role of stem cells for neuropathic pain disorders. Neurosurg Focus.

[56] Han YH, Kim KH, Abdi S (2019). Stem cell therapy in pain medicine. Korean J Pain.

[57] Orozco L, Soler R, Morera C (2011). Intervertebral disc repair by autologous mesenchymal bone marrow cells: a pilot study. Transplantation.

[58] Dadon-Nachum M, Sadan O, Srugo I (2011). Differentiated mesenchymal stem cells for sciatic nerve injury. Stem Cell Rev Rep.

[59] Premaratne GU, Ma LP, Fujita M (2011). Stromal vascular fraction transplantation as an alternative therapy for ischemic heart failure: anti-inflammatory role. J Cardiothorac Surg.

[60] Fain JN, Madan AK, Hiler ML (2004). Comparison of the release of adipokines by adipose tissue, adipose tissue matrix, and adipocytes from visceral and subcutaneous abdominal adipose tissues of obese humans. Endocrinology.

[61] Naruse K, Sato J, Funakubo M (2011). Transplantation of bone marrow-derived mononuclear cells improves mechanical hyperalgesia, cold allodynia and nerve function in diabetic neuropathy. PLoS ONE.

[62] Siniscalco D, Giordano C, Galderisi U (2011). Long-lasting effects of human mesenchymal stem cell systemic administration on pain-like behaviors, cellular, and biomolecular modifications in neuropathic mice. Front Integr Neurosci.

[63] Guo W, Wang H, Zou S (2011). Bone marrow stromal cells produce long-term pain relief in rat models of persistent pain. Stem Cells.

[64] Ichim TE, Solano F, Lara F (2010). Feasibility of combination allogeneic stem cell therapy for spinal cord injury: a case report. Int Arch Med.

[65] Vickers ER, Karsten E, Flood J (2014). A preliminary report on stem cell therapy for neuropathic pain in humans. J Pain Res.

[66] Wagner W, Ho AD, Zenke M (2010). Different facets of aging in human mesenchymal stem cells. Tissue Eng Part B Rev.

[67] Vacanti V, Kong E, Suzuki G (2005). Phenotypic changes of adult porcine mesenchymal stem cells induced by prolonged passaging in culture. J Cell Physiol.

[68] Franchi S, Castelli M, Amodeo G (2014). Adult stem cell as new advanced therapy for experimental neuropathic pain treatment. Biomed Res Int.

[69] Sorkin LS, Yaksh TL (2009). Behavioral models of pain states evoked by physical injury to the peripheral nerve. Neurotherapeutics.

[70] Leung A, Fallah A, Shukla S (2014). Transcutaneous magnetic stimulation (TMS) in alleviating post-traumatic peripheral neuropathic pain States: a case series. Pain Med.

[71] Barker AT, Jalinous R, Freeston IL (1985). Non-invasive magnetic stimulation of human motor cortex. Lancet.

[72] Pridmore S, Oberoi G (2000). Transcranial magnetic stimulation applications and potential use in chronic pain: studies in waiting. J Neurol Sci.

[73] Lefaucheur JP, Ayache SS, Sorel M (2012). Analgesic effects of repetitive transcranial magnetic stimulation of the motor cortex in neuropathic pain: influence of theta burst stimulation priming. Eur J Pain.

[74] Yoo WK, You SH, Ko MH (2008). High frequency rTMS modulation of the sensorimotor networks: behavioral changes and fMRI correlates. Neuroimage.

[75] Marlow NM, Bonilha HS, Short EB (2013). Efficacy of transcranial direct current stimulation and repetitive transcranial magnetic stimulation for treating fibromyalgia syndrome: a systematic review. Pain Pract.

[76] O'Connell NE, Marston L, Spencer S, DeSouza LH, Wand BM. Non-invasive brain stimulation techniques for chronic pain. Cochrane Database Syst Rev. 2018;3(3):CD008208. Published 2018 Mar 16. 10.1002/14651858.CD008208.pub4. 10.1002/14651858.CD008208.pub4 PMC7039253 29547226

[77] O'Connell NE, Wand BM, Marston L (2011). Non-invasive brain stimulation techniques for chronic pain. A report of a Cochrane systematic review and meta-analysis. Eur J Phys Rehabil Med.

[78] Rao VP, Satyarengga M, Lamos EM (2021). The use of transcutaneous magnetic stimulation to treat painful diabetic neuropathy. J Diabetes Sci Technol.

[79] Young NA, Sharma M, Deogaonkar M (2014). Transcranial magnetic stimulation for chronic pain. Neurosurg Clin N Am.

[80] Guindon J, Hohmann AG (2008). Cannabinoid CB2 receptors: a therapeutic target for the treatment of inflammatory and neuropathic pain. Br J Pharmacol.

[81] Galiegue S, Mary S, Marchand J (1995). Expression of central and peripheral cannabinoid receptors in human immune tissues and leukocyte subpopulations. Eur J Biochem.

[82] Manzanares J, Cabanero D, Puente N (2018). Role of the endocannabinoid system in drug addiction. Biochem Pharmacol.

[83] Pertwee RG (2008). The diverse CB1 and CB2 receptor pharmacology of three plant cannabinoids: delta9-tetrahydrocannabinol, cannabidiol and delta9- tetrahydrocannabivarin. Br J Pharmacol.

[84] Wehbi VL, Tasken K (2016). Molecular mechanisms for cAMP-mediated immunoregulation in T cells - role of anchored protein kinase A signaling units. Front Immunol.

[85] Ghosh K, Zhang GF, Chen H (2022). Cannabinoid CB2 receptors are upregulated via bivalent histone modifications and control primary afferent input to the spinal cord in neuropathic pain. J Biol Chem.

[86] Sainsbury B, Bloxham J, Pour MH (2021). Efficacy of cannabis-based medications compared to placebo for the treatment of chronic neuropathic pain: a systematic review with meta- analysis. J Dent Anesth Pain Med.

[87] Almog S, Aharon-Peretz J, Vulfsons S (2020). The pharmacokinetics, efficacy, and safety of a novel selective-dose cannabis inhaler in patients with chronic pain: a randomized, double-blinded, placebo-controlled trial. Eur J Pain.

[88] Brown JD. Potential Adverse Drug Events with Tetrahydrocannabinol (THC) Due to Drug-Drug Interactions. J Clin Med. 2020;9(4):919. Published 2020 Mar 27. 10.3390/jcm9040919. 10.3390/jcm9040919 PMC7231229 32230864

[89] van Hecke O, Austin SK, Khan RA (2014). Neuropathic pain in the general population: a systematic review of epidemiological studies. Pain.

[90] Fornasari D (2017). Pharmacotherapy for neuropathic pain: a review. Pain Ther.

[91] Yu K, Niu X, He B. Neuromodulation Management of Chronic Neuropathic Pain in The Central Nervous system. Adv Funct Mater. 2020;30(37):1908999. 10.1002/adfm.201908999. 10.1002/adfm.201908999 PMC8323399 34335132

[92] Hofmeister M, Memedovich A, Brown S (2020). Effectiveness of neurostimulation technologies for the management of chronic pain: a systematic review. Neuromodulation.

[93] Baig MZ, Abdullah UEH, Muhammad A (2021). Use of platelet-rich plasma in treating low back pain: a review of the current literature. Asian Spine J.

[94] Shen L, Yuan T, Chen S (2017). The temporal effect of platelet-rich plasma on pain and physical function in the treatment of knee osteoarthritis: systematic review and meta- analysis of randomized controlled trials. J Orthop Surg Res.

[95] Bhatia R, Chopra G. Efficacy of Platelet Rich Plasma via Lumbar Epidural Route in Chronic Prolapsed Intervertebral Disc Patients-A Pilot Study. J Clin Diagn Res. 2016;10(9):UC05-UC07. 10.7860/JCDR/2016/21863.8482. 10.7860/JCDR/2016/21863.8482 PMC5072053 27790553

[96] Mohammed S, Yu J (2018). Platelet-rich plasma injections: an emerging therapy for chronic discogenic low back pain. J Spine Surg.

[97] Hussein M, Hussein T (2016). Effect of autologous platelet leukocyte rich plasma injections on atrophied lumbar multifidus muscle in low back pain patients with monosegmental degenerative disc disease. Sicot j.

[98] Navani A, Li G, Chrystal J (2017). Platelet rich plasma in musculoskeletal pathology: a necessary rescue or a lost cause?. Pain Physician.

[99] Krsticevic M, Jeric M, Dosenovic S (2017). Proliferative injection therapy for osteoarthritis: a systematic review. Int Orthop.

[100] Rahimzadeh P, Imani F, Faiz SHR (2018). The effects of injecting intra-articular platelet-rich plasma or prolotherapy on pain score and function in knee osteoarthritis. Clin Interv Aging.

[101] Reeves KD, Sit RW, Rabago DP (2016). Dextrose prolotherapy: a narrative review of basic science, clinical research, and best treatment recommendations. Phys Med Rehabil Clin N Am.

[102] Sánchez-Robles EM, Girón R, Paniagua N, Rodríguez-Rivera C, Pascual D, Goicoechea C. Monoclonal Antibodies for Chronic Pain Treatment: Present and Future. Int J Mol Sci. 2021;22(19):10325. Published 2021 Sep 25. 10.3390/ijms221910325. 10.3390/ijms221910325 PMC8508878 34638667

[103] Zhang FF, Morioka N, Harano S (2016). Perineural expression of high-mobility group box-1 contributes to long-lasting mechanical hypersensitivity via matrix metalloprotease-9 up- regulation in mice with painful peripheral neuropathy. J Neurochem.

[104] Abdiche YN, Malashock DS, Pons J (2008). Probing the binding mechanism and affinity of tanezumab, a recombinant humanized anti-NGF monoclonal antibody, using a repertoire of biosensors. Protein Sci.

[105] Bannwarth B, Kostine M (2014). Targeting nerve growth factor (NGF) for pain management: what does the future hold for NGF antagonists?. Drugs.

[106] Watson JJ, Allen SJ, Dawbarn D (2008). Targeting nerve growth factor in pain: what is the therapeutic potential?. BioDrugs.

[107] Campbell JN, Meyer RA (2006). Mechanisms of neuropathic pain. Neuron.

[108] Leung L, Cahill CM (2010). TNF-alpha and neuropathic pain–a review. J Neuroinflammation.

[109] Hess A, Axmann R, Rech J (2011). Blockade of TNF-alpha rapidly inhibits pain responses in the central nervous system. Proc Natl Acad Sci USA.

[110] Maini RN, Breedveld FC, Kalden JR (1998). Therapeutic efficacy of multiple intravenous infusions of anti-tumor necrosis factor alpha monoclonal antibody combined with low- dose weekly methotrexate in rheumatoid arthritis. Arthritis Rheum.

[111] Dirckx M, Groeneweg G, Wesseldijk F (2013). Report of a preliminary discontinued double- blind, randomized, placebo-controlled trial of the anti-TNF-alpha chimeric monoclonal antibody infliximab in complex regional pain syndrome. Pain Pract.

[112] Yeh JF, Akinci A, Al Shaker M (2017). Monoclonal antibodies for chronic pain: a practical review of mechanisms and clinical applications. Mol Pain.

[113] Bannwarth B, Kostine M (2015). Biologics in the treatment of chronic pain: a new era of therapy?. Clin Pharmacol Ther.

